# Sequential dengue virus infections detected in active and passive surveillance programs in Thailand, 1994–2010

**DOI:** 10.1186/s12889-015-1590-z

**Published:** 2015-03-14

**Authors:** Piraya Bhoomiboonchoo, Ananda Nisalak, Natkamol Chansatiporn, In-Kyu Yoon, Siripen Kalayanarooj, Mathuros Thipayamongkolgul, Timothy Endy, Alan L Rothman, Sharone Green, Anon Srikiatkhachorn, Darunee Buddhari, Mammen P Mammen, Robert V Gibbons

**Affiliations:** Department of Virology, Armed Forces Research Institute of Medical Sciences, Bangkok, Thailand; Faculty of Public Health, Mahidol University, Bangkok, Thailand; Queen Sirikit National Institute of Child Health, Bangkok, Thailand; Department of Infectious Diseases, State University of New York, Syracuse, NY USA; University of Rhode Island, Providence, RI USA; Division of Infectious Diseases and Immunology, University of Massachusetts Medical School, Worcester, MA USA; Vical, Incorporated, San Diego, CA USA; US Army Institute of Surgical Research, San Antonio, TX USA

## Abstract

**Background:**

The effect of prior dengue virus (DENV) exposure on subsequent heterologous infection can be beneficial or detrimental depending on many factors including timing of infection. We sought to evaluate this effect by examining a large database of DENV infections captured by both active and passive surveillance encompassing a wide clinical spectrum of disease.

**Methods:**

We evaluated datasets from 17 years of hospital-based passive surveillance and nine years of cohort studies, including clinical and subclinical DENV infections, to assess the outcomes of sequential heterologous infections. Chi square or Fisher’s exact test was used to compare proportions of infection outcomes such as disease severity; ANOVA was used for continuous variables. Multivariate logistic regression was used to assess risk factors for infection outcomes.

**Results:**

Of 38,740 DENV infections, two or more infections were detected in 502 individuals; 14 had three infections. The mean ages at the time of the first and second detected infections were 7.6 ± 3.0 and 11.2 ± 3.0 years. The shortest time between sequential infections was 66 days. A longer time interval between sequential infections was associated with dengue hemorrhagic fever (DHF) in the second detected infection (OR 1.3, 95% CI 1.2-1.4). All possible sequential serotype pairs were observed among 201 subjects with DHF at the second detected infection, except DENV-4 followed by DENV-3. Among DENV infections detected in cohort subjects by active study surveillance and subsequent non-study hospital-based passive surveillance, hospitalization at the first detected infection increased the likelihood of hospitalization at the second detected infection.

**Conclusions:**

Increasing time between sequential DENV infections was associated with greater severity of the second detected infection, supporting the role of heterotypic immunity in both protection and enhancement. Hospitalization was positively associated between the first and second detected infections, suggesting a possible predisposition in some individuals to more severe dengue disease.

**Electronic supplementary material:**

The online version of this article (doi:10.1186/s12889-015-1590-z) contains supplementary material, which is available to authorized users.

## Background

Dengue is a globally important, re-emerging infectious disease caused by one of four dengue virus serotypes (DENV-1 to DENV4) with a high degree of antigenic cross-reactivity. It is estimated that 390 million infections occur annually, with approximately 96 million resulting in clinically apparent disease [1]. DENV infections can lead to diverse outcomes, including subclinical infection, clinically non-specific illness, dengue fever (DF), and dengue hemorrhagic fever (DHF).

Many studies have shown that the risk of DHF in non-infant patients is greater when an initial DENV infection is followed by a second infection with a different serotype [2-7]. All possible orders of infecting serotypes have been documented in patients with DHF except DENV-4 followed by DENV-1 or DENV-3 [8]. In some populations, reports indicate that DHF occurs more frequently with DENV-2 or DENV-3 infections in DENV-1 exposed individuals [9,10]. One mechanism underlying this observation has been postulated to be antibody-dependent enhancement (ADE) during the second infection mediated by non-protective heterotypic antibodies arising from the first infection. However, the timing of the second infection seems to be important since some degree of short-term protection may be conferred against subsequent heterologous infection by the preceding infection [11]. In a population model of children hospitalized with dengue in Bangkok, Thailand, the length of this short-term heterologous protection was estimated to be one to three years [12]. Longer intervals between heterologous infections seem to increase susceptibility to DHF. An evaluation of dengue cases from outbreaks in Cuba in 1981 and 1997 suggest that a longer period between infections increases the risk of DHF [13]. In an analysis of repeat DENV infections from a prospective cohort study of children in Kamphaeng Phet, Thailand, the ratio of symptomatic to subclinical infections was found to be higher when the time from first to second infection was longer [14].

Some studies have suggested that sequential infection with two different serotypes may induce sufficient cross-immunity to confer some degree of protection from a third or fourth serotype. Primate studies have suggested that multivalent neutralizing antibodies after two DENV infections reduce the risk of detectable viremia from subsequent heterologous infection [15-18]. Among thousands of children hospitalized with dengue in Bangkok, Thailand, the number of known third and fourth infections was found to be less than the number of known second infections indicating some level of multivalent protection after two heterologous infections [8]. Interestingly, in this same population of hospitalized children, the ratio of DHF to DF with known second infections was no different than with known third or fourth infections. In an analysis of a prospective cohort from Iquitos, Peru, presumed second infections were more likely to be symptomatic rather than subclinical as compared to third or fourth infections [19].

Altogether, these observations highlight the intricate interplay between host immunity and sequential DENV infections and suggest that the time between consecutive infections is an important factor in infection outcome. In Bangkok and Kamphaeng Phet, Thailand, where dengue is hyperendemic, our group has conducted prospective cohort studies, hospital-based dengue studies and public health diagnostic testing for several decades. These have resulted in a large database containing thousands of DENV infections. Repeat infections in some of these datasets have been analyzed previously and published elsewhere [8,14]. Data used in these prior analyses were partitioned according to data source to avoid study biases. In contrast, the current study combines all these different datasets into a larger single dataset from which previously unidentified repeat infections could be detected, potentially yielding a wider clinical spectrum of repeat infections and allowing detection of sequential serotype pairs which would not otherwise have been detected.

## Methods

### Ethics statement

The retrieval and analysis of coded pre-existing data in this study was approved by the Walter Reed Army Institute of Research (WRAIR) Institutional Review Board (IRB), Queen Sirikit National Institute of Child Health (QSNICH) IRB and Kamphaeng Phet Provincial Hospital (KPPPH). Public health samples from passive surveillance were originally collected at QSNICH and KPPPH, and data were analyzed anonymously. Research study samples were originally collected from QSNICH, KPPPH, and Kamphaeng Phet (KPP) province according to their respective approved study protocols, and data were analyzed according to those protocols. The original study protocols were approved by the IRBs of WRAIR, University of Massachusetts Medical School, the Thai Ministry of Public Health (MOPH), and QSNICH as described in earlier publications.

### Datasets and locations

Datasets from three types of samples were used to identify repeat infections in the current study: (1) public health samples from passive surveillance of hospitalized DENV infections at QSNICH and KPPPH; (2) research samples from hospital-based dengue studies at QSNICH and KPPPH, and (3) research samples from prospective cohort studies in KPP province. For research samples, written informed consents were obtained from the parent/guardian of study subjects. QSNICH is a 420-bed tertiary care pediatric hospital located in Bangkok, Thailand that serves as the MOPH dengue referral center for Bangkok. KPPPH is a 334-bed facility that serves as the MOPH referral hospital for KPP province, a rural area of over 700,000 people located approximately 350 km north of Bangkok. Procedures used to generate each dataset have been detailed elsewhere and are briefly described here.

### Public health samples

Acute and convalescent blood samples from passive surveillance of clinically suspected dengue inpatients at QSNICH and KPPPH were tested (as described in the “Laboratory Dengue Assays” section) for evidence of DENV infection at the Armed Forces Research Institute of Medical Sciences (AFRIMS) for public health purposes [8]. Testing was requested at the discretion of the health care provider for clinically suspected cases. No specific criteria were required either for hospitalization or for dengue testing; these were based on the clinical judgment of the health care provider. Patients admitted to QSNICH were ≤15 years old; patients at KPPPH could be of any age. Clinical classification was based on World Health Organization (WHO) guidelines applicable at the time of infection. Dengue cases admitted from 1994 to 2010 were used for the current analysis.

### Hospital-based dengue studies

Hospital-based prospective dengue studies were conducted at KPPPH from 1994–97 and at QSNICH from 1994–2002 [20,21]. Children ≤15 years old who presented to the hospital outpatient department with fever ≤3 days duration without an obvious source of infection were enrolled and followed prospectively in the hospital. Acute and convalescent blood samples were tested (as described in the “Laboratory Dengue Assays” section) for DENV infection at AFRIMS. Clinical classification was based on World Health Organization (WHO) criteria applicable at the time of infection. Data from dengue cases from 1994 to 2002 were used for the current analysis.

### Prospective cohort studies

Two longitudinal prospective cohort studies were conducted in Kamphaeng Phet, Thailand from 1998–2002 (called KPS1) and from 2004–2007 (called KPS2) [20,21]. Approximately 2000 primary school children were followed for acute febrile episodes during the presumed peak DENV transmission season (Jun-Nov/Dec) using active school absence-based surveillance. Acute and convalescent blood samples from acute febrile episodes were tested (as described in the “Laboratory Dengue Assays” section) for DENV infection at AFRIMS. Quarterly (for KPS1) or pre- and post-surveillance season (for KPS2) blood collections were tested for evidence of seroconversion to DENV. Subclinical infection was defined as ≥ four-fold rise in dengue hemagglutination inhibition (HAI) titer between scheduled blood collections [22] confirmed by dengue plaque reduction neutralization test (PRNT) [23], but without an acute symptomatic DENV infection detected during the intervening period. Symptomatic infection was defined as an acute febrile episode caused by laboratory-confirmed DENV infection. Seroconversion during the non-surveillance period could not be classified as subclinical or symptomatic infection since active surveillance was not performed during that period. Symptomatic infection was further categorized as non-hospitalized DF, hospitalized DF (hDF) and hospitalized DHF. Clinical classification was based on WHO 1997 guidelines [24]. Data from subclinical and symptomatic DENV infections from 1998 to 2007 were used for the current analysis.

### Laboratory dengue assays

Acute blood samples were tested by viral isolation [25] and/or hemi-nested reverse transcriptase polymerase chain reaction (RT-PCR) [26-29]. Acute and convalescent blood samples were tested by DENV IgM/IgG enzyme-linked immunosorbent assay (ELISA) [30]. If anti-dengue IgM was ≥40 units and anti-dengue IgM:IgG ratio was ≥1.8, the infection was considered as “primary”. An infection was considered “secondary” if anti-dengue IgM was ≥40 units and anti-dengue IgM:IgG ratio was <1.8, or, if anti-dengue IgM was <40, there was ≥ two-fold increase in anti-dengue IgG with an absolute value of ≥100 units for acute/convalescent sample pairs. In the cohort studies, paired samples from periodic scheduled blood collections were tested by dengue HAI and, if positive for seroconversion based on a ≥ four-fold rise in titer, were confirmed by dengue PRNT.

### Data management and terminology

Data managers independent of study investigators identified repeat DENV infections occurring in the same individual by name and birth dates from the relevant datasets. The data was then anonymized and provided to study investigators for further analysis.

“Primary” infection was presumed to be the first DENV infection in an individual and was defined serologically as described above. “Secondary” infection was considered to be any subsequent infection after a primary infection and was defined serologically. “First, second, third, and fourth” infections refer to the order of sequential infections in an individual whether or not the infection was actually detected. Second, third and fourth infections were all considered to be “secondary” infections. “Infection one, two, and three” refer to the order of sequential infections actually detected in an individual during the study period (i.e., infection one was the first detected infection, etc.).

### Statistical methods and data analysis

Demographic characteristics were described and the time between sequential infections was calculated by subtracting dates of sample collection. For subclinical infections (only available from the cohort studies), the midpoint time between scheduled blood collections was used as the time of infection. Chi-square test and odds ratios (ORs) with 95% confidence intervals (CIs) were used to compare proportions in clinical severity measures (e.g., hospitalization versus non-hospitalization, DHF versus non-DHF). Fisher’s exact test was used to compare DHF:DF ratios among different sequential serotype pairs. For continuous variables, one-way ANOVA followed by Bonferroni multiple comparison correction was used. Since the different datasets used for the current evaluation came from studies and surveillance programs with different methodologies, quantitative analysis of risk factors for clinical severity was limited to DENV infections in cohort subjects detected during active cohort study surveillance and subsequent non-study hospital-based passive surveillance (hereafter referred to as “cohort extended dataset”). The duration of hospital-based passive surveillance used in the cohort extended dataset was limited to five years after infection one to minimize unequal chances of detecting sequential infections due to this extended surveillance period. Multivariate binary logistic regression was used to assess risk factors associated with clinical severity. Data were analyzed using SPSS for Windows (version 19) and Stata/MP 11.1 for Windows.

## Results

Data from 38,740 laboratory-confirmed DENV infections was available for evaluation. Among these infections, we identified 502 individuals who had repeat infections comprising 1,018 sequential DENV infections; 488 individuals had two detected infections and 14 had three detected infections. Their demographic, serological, and clinical characteristics are shown in Table 1. Details of the 14 individuals with three documented DENV infections are given in Table 2. Because the number of such individuals was small, infection three was excluded from subsequent analyses.

**Table 1:** **Demographic, serologic, and clinical characteristics of individuals with repeat dengue virus infections**

	**All data (n = 502 individuals)** ^**a**^	**Cohort extended dataset (n = 205)**
**Gender**		
Female	252	100
Male	237	95
Unknown	13	10
**Age**		
At infection one (mean years ± SD)	7.6 ± 3.0	9.0 ± 1.7
At infection two (mean years ± SD)	11.2 ± 3.0	11.2 ± 2.0
**Serologic response at infection one** ^**b**^		
Primary	84	6
Secondary	263	59
Unknown (symptomatic)	15	-
Unknown (Subclinical/nonhospitalized)	56	56
Unknown (Subclinical)	84	84
**Clinical category at infection one** ^**c**^		
Symp. (DHF)	201	24
Symp. (DHF/hospitalized DF)	16	3
Symp. (hospitalized DF)	123	16
Symp. (non-hospitalized DF)	19	19
Symp. (hospitalized/non-hospitalized DF)	3	3
Subclinical/non-hospitalized	56	56
Subclinical	84	84
**Clinical category at infection two** ^**c**^		
Symp. (DHF)	201	17
Symp. (DHF/hospitalized DF)	13	-
Symp. (hospitalized DF)	112	16
Symp. (non-hospitalized DF)	33	29
Symp. (hospitalized/non-hospitalized DF)	1	1
Subclinical/non-hospitalized	42	42
Subclinical	100	100

^a^488 individuals had two detected infections; 14 had three detected infections.

^b^The serologic response could not be categorized in 155 individuals; 140 could not be categorized because they were either subclinical (n = 84) or had seroconversion outside the active surveillance period in cohort studies (n = 56); 15 were symptomatic but had inadequate samples to determine serologic response.

^c^Subclinical infections in cohort studies were identified by ≥ four-fold rise in dengue hemagglutination inhibition (HAI) titers without detection of symptomatic infection during the intervening surveillance period. Infections captured outside the active study surveillance period by HAI without a recognized hospitalization were marked as ‘subclinical/non-hospitalized’. Hospitalized cases that did not have sufficient clinical information for clinical categorization were marked ‘DHF/hospitalized DF.’ Four infections categorized as DF but with uncertain hospitalization status were marked as ‘hospitalized/non-hospitalized DF’.

**Table 2:** **Data of individuals with three detected infections**

		**Age at infection**			**Serotype** ^**a**^			**Serologic response** ^**b**^			**Clinical category** ^**c**^		
	**Infection order**	**1**	**2**	**3**	**1**	**2**	**3**	**1**	**2**	**3**	**1**	**2**	**3**
**Indiv.**	**Titers > 10** ^**d**^												
1	0	8.7	12.8	14.9	ND	NEG	DEN4	ND	S	S	SubC/nhDF	DHF	DHF
2	0	8.9	10.9	12.2	ND	ND	ND	ND	ND	ND	SubC	SubC	SubC
3	ND	10.5	10.8	10.9	NEG	DEN1	NEG	S	S	S	nhDF/hDF	hDF	hDF
4	0	7.7	8.7	11.0	ND	ND	DEN2	ND	ND	S	SubC/nhDF	SubC/nhDF	nhDF
5	4	7.5	8.5	10.5	ND	ND	ND	ND	ND	ND	SubC	SubC	SubC
6	0	7.9	9.2	12.2	ND	ND	ND	ND	ND	ND	SubC/nhDF	SubC	SubC
7	0	7.9	9.4	12.8	DEN1	NEG	ND	S	S	ND	DHF	nhDF	SubC
8	4	9.0	12.3	12.9	ND	ND	DEN2	ND	ND	S	SubC	SubC	DHF
9	ND	5.9	9.3	10.2	NEG	ND	ND	S	ND	ND	DHF	SubC	SubC
10	4	7.8	9.0	10.3	ND	ND	ND	ND	ND	ND	SubC/nhDF	SubC	SubC
11	ND	9.1	11.1	11.8	DEN1	DEN3	ND	S	S	ND	hDF	nhDF	SubC/nhDF
12	0	7.8	10.8	12.3	ND	ND	ND	ND	ND	ND	SubC/nhDF	SubC/nhDF	SubC
13	0	7.8	8.8	10.1	ND	ND	DEN2	ND	ND	S	SubC/nhDF	SubC/nhDF	nhDF
14	ND	5.3	8.7	9.8	NEG	DEN2	ND	S	S	ND	DHF	nhDF	SubC

^a^Dengue virus (DENV) serotype detected by RT-PCR or viral culture. NEG = no virus detected. ND = RT-PCR not performed (no symptomatic infection occurred).

^b^S = secondary response. ND = not determined (no symptomatic infection occurred).

^c^SubC = subclinical DENV infection (captured by four-fold rise in dengue hemagglutination inhibition (HAI) titers without identified acute symptomatic DENV infection); SubC/nhDF = subclinical/non-hospitalized dengue fever (DF) (infections captured by four-fold rise in HAI titers outside the active study surveillance period; see Methods for detailed explanation); nhDF = non-hospitalized DF; hDF = hospitalized DF.

^d^Number of DENV serotypes with HAI titer >10 prior to infection one.

**Figure 1:**
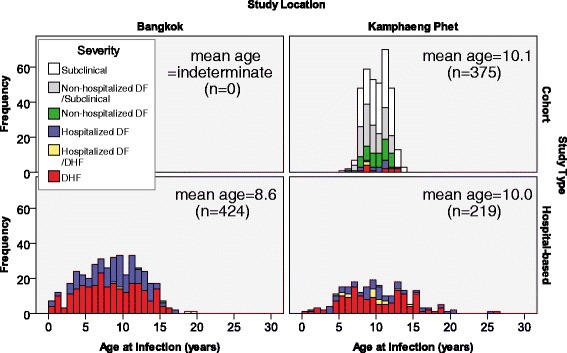
**Age at time of infection by location.** Data used in this study were collected from two locations: Kamphaeng Phet and Bangkok, Thailand. Age distributions of infections detected in each dataset for each location are shown.

Sixty-three percent (643/1,018) of repeat infections were detected from public health samples or hospital-based dengue studies through passive surveillance; 424 were from QSNICH and 219 were from KPPPH. The remaining 375 infections were detected from prospective cohort studies in KPP through active and passive surveillance. Mean ages at infection one and infection two captured during cohort studies versus hospital-based passive surveillance are shown in Figure 1. As expected, significant age differences were found among study/surveillance types and locations. DENV infections were clinically categorized into four groups: subclinical, non-hospitalized DF (nhDF), hospitalized DF (hDF), and DHF (all of which were hospitalized). The latter three groups were considered to be symptomatic infections. Among 29 hospitalized infections (16 infection one’s; 13 infection two’s), data was not available to determine whether these were hDF or DHF. Ninety-eight infections from cohort studies detected by dengue HAI seroconversion outside the active study surveillance period had indeterminate serotype and clinical severity. Infections that could not be clinically categorized were removed from analyses that required this information (e.g., DHF versus DF, symptomatic versus subclinical).

### Serologic response

**Figure 2:**
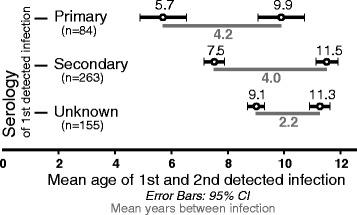
**Mean age at infection one and infection two (with 95% CIs) along with the mean time interval between infections grouped by serologic response at infection one.**

Data on the acute serologic response (i.e., primary versus secondary) at infection one was available for 347 individuals with symptomatic infections; 15 individuals with symptomatic infection at infection one had insufficient samples to determine serological response. Of these 347 individuals, 84 (24.2%) demonstrated a primary serologic response and 263 (75.8%) a secondary response. The mean age of individuals with a secondary response at infection one was 7.5 ± 2.9 years, which was between the mean age with a primary response at infection one (5.7 ± 3.8 years; p < 0.001) and the corresponding infection two (9.9 ± 3.7 years; p < 0.001, Figure 2).

### Sequential serotype pairs

All possible pairs of sequential infecting serotypes were documented except for DENV-4 followed by DENV-3. DHF in infection two was documented for all these serotype pairs including DENV-4 followed by DENV-1 (Table 3). No sequential infections with the same serotype were detected. Thirteen individuals for whom DHF/DF classification in infection two was not available (marked as DHF/hospitalized DF in Table 1) were not included in Table 3. There was no significant difference in the ratio of DHF to DF among the different sequential serotype pairs regardless of the serologic responses. However, statistical power was limited by the small numbers within each cell.

**Table 3:** **Ratio of dengue hemorrhagic fever to dengue fever (DHF:DF) at infection two in individuals with two symptomatic infections according to sequential serotype pair and serologic response at infection one**

**Serotype at infection one**	**Serological response at infection one**	**DHF:DF ratio**				
		**Serotype at infection two**				
		**DENV-1**	**DENV-2**	**DENV-3**	**DENV-4**	**Any** ^**a**^
DENV-1	Primary	-	9.0 (9:1)	Ind. (4:0)	2.0 (8:4)	3.3 (23:7)
	Secondary	-	1.2 (13:11)	1.3 (5:4)	0.6 (3:5)	1.3 (31:23)
	All^b^	-	1.8 (24:13)	2.3 (9:4)	1.1 (11:10)	1.7 (58:34)
DENV-2	Primary	2.0 (2:1)	-	Ind. (1:0)	1.0 (1:1)	2 (6:3)
	Secondary	5.0 (5:1)	-	4.0 (4:1)	5.0 (5:1)	2.6 (21:8)
	All^b^	3.5 (7:2)	-	5.0 (5:1)	3.0 (6:2)	2.3 (27:12)
DENV-3	Primary	3.0 (3:1)	1.0 (2:2)	-	1.0 (1:1)	1.1 (9:8)
	Secondary	2.0 (4:2)	3.5 (7:2)	-	0.6 (3:5)	1.6 (19:12)
	All^b^	2.3 (7:3)	2.3 (9:4)	-	0.7 (4:6)	1.4 (28:20)
DENV-4	Primary	-	-	-	-	-
	Secondary	0.5 (1:2)	0.5 (1:2)	-	-	0.7 (4:6)
	All^b^	0.5 (1:2)	1.0 (2:2)	-	-	0.8 (5:6)
Any^a^	Primary	2.7 (8:3)	3.6 (18:5)	Ind. (6:0)	1.6 (11:7)	2.2 (55:25)
	Secondary	1.2 (16:13)	1.7 (38:22)	1.1 (10:9)	1.2 (20:17)	1.4 (127:88)
	All^b^	1.5 (24:16)	2.3 (63:28)	1.8 (16:9)	1.3 (32:25)	1.6 (192:118)

A total of 323 of 502 sequential dengue virus (DENV) infections were symptomatic at both infection one and two. Of these 323, 13 without DHF/DF classification at infection two (marked as DHF/hospitalized DF in Table 1) were not included in this table. Statistical power was limited by the small numbers within each cell.

^a^Includes any case captured regardless of knowing or not knowing the infecting serotype.

^b^Includes all cases captured regardless of serologic response at infection one. Therefore, ones with unknown response are also included.

Ind = indeterminate.

### Time intervals between infections

The shortest time between any two sequential DENV infections was 66 days (Table 4). The mean interval between infection one and infection two was shorter when infection two was subclinical than when infection two was symptomatic (1.9 vs. 4.1 years; p < 0.001). Every additional year between sequential infections increased the risk of hospitalization; the crude OR increased by 1.3 (95% CI 0.7-2.3), 1.9 (95% CI 1.5-2.4), and 3.2 (95% CI 2.0-4.9) times each year when infection one had primary, secondary, and unknown serologic response, respectively, but only reached significance for the latter two (both p < 0.001). Subclinical infections were only available from cohort studies, whereas most of the symptomatic cases were captured from hospital-based passive surveillance.

**Table 4:** **Time interval between sequential dengue virus infections by clinical category at infection one and two**

		**Interval between infections (yrs)**							
**Clinical category** ^**a**^		**All data (n = 502)**				**Cohort extended dataset** ^**c**^ **(n = 195)**			
Infection one	Infection two	N	Mean	Min	Max	N	Mean	Min	Max
Any	Any	502	3.45	0.18	12.57	195	1.85	.27	4.96
Subclinical^b^	Subclinical^b^	45	1.74	0.78	3.25	45	1.74	0.78	3.25
Subclinical^b^	Symptomatic	26	3.03	0.65	12.26	21	1.84	0.65	4.22
Symptomatic	Subclinical^b^	23	2.15	0.77	4.96	23	2.15	0.77	4.96
Symptomatic	Symptomatic	323	4.27	0.18	12.57	21	2.35	0.27	4.28

^a^Clinical category at infection one and two.

^b^Subclinical infections were only captured from cohort studies. Symptomatic infections include all symptomatic infections from Bangkok and Kamphaeng Phet. 56 infection one’s and 42 infection two’s were unable to be clinically categorized as symptomatic or subclinical due to occurrence out of the active study surveillance period and not seeking hospital care (listed as ‘Any’ in the table).

^c^Only infections in cohort subjects were included in this subgroup; includes infections detected from non-study hospital-basedpassive surveillance within five years after infection one.

**Figure 3:**
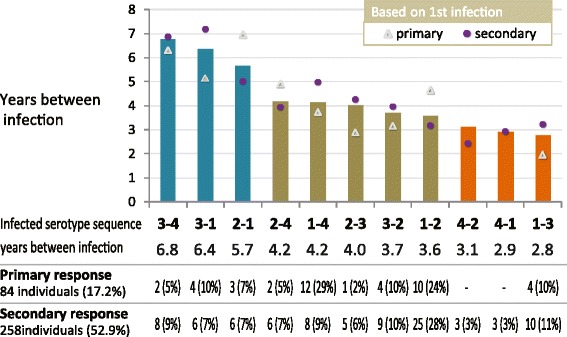
**Time between sequential infections versus each sequential serotype pair (only individuals with known serotypes for both sequential infections are included).** Time between sequential infections (N = 187) were plotted in ordinal. Numbers with known serologic response at infection one are shown.

The number of individuals with each sequential serotype pair was relatively small, ranging from three with DENV-4 followed by DENV-1, to 37 with DENV-1 followed by DENV-2. The intervals between infection one and infection two were compared among the different serotype pairs (Figure 3). DENV-3 followed by DENV-4 had the longest interval (6.8 ± 1.8 years), which was significantly longer than DENV-1 followed by DENV-3 (2.8 ± 1.6 years, p = 0.001), DENV-1 followed by DENV-2 (3.6 ± 1.8 years, p = 0.002), and DENV-3 followed by DENV-2 (3.7 ± 2.2 years, p = 0.043). The other serotype pairs with significant differences were DENV-3 followed by DENV-1 (6.4 ± 2.7 years) compared to DENV-1 followed by DENV-3 (2.8 ± 1.6 years, p = 0.003) and DENV-1 followed by DENV-2 (3.6 ± 1.8 years, p = 0.015). These differences in time intervals may simply reflect the serotype circulation patterns in the general population. The mean time between infection one and infection two was not significantly different based on whether infection one was associated with a primary serologic response (4.2 ± 2.5 years) or a secondary response (3.9 ± 2.4 years); p = 0.392.

### Analysis of cohort extended dataset

To determine relationships between characteristics of the sequential DENV infections and clinical severity at infection two, we limited analysis to infections in cohort subjects only, from both active study surveillance and subsequent non-study hospital-based passive surveillance. Of the 502 individuals with repeat detected DENV infections, 158 had their infections captured solely from cohort study surveillance. When subsequent non-study hospital-based passive surveillance was included an additional 37 cohort subjects with repeat infections were identified. Gender, mean age at each infection and clinical categorization are shown in Table 1. A comparison of characteristics of these 158 and 37 individuals are presented in the Additional file 1.

#### Univariate analysis of association with clinical category

Comparisons were done for hospitalized versus non-hospitalized dengue and DHF versus non-DHF. The risk of hospitalization at infection two was increased by 1.8 (95% CI 1.21-2.67) for each additional year between sequential infections. Hospitalization at infection one also increased the risk of hospitalization at infection two (OR 4.58, 95% CI 1.37-15.24) while having DHF at infection one did not. Age at infection one was not associated with hospitalization at infection two. Similar relationships were found when examining risk for DHF versus non-DHF; odds ratios are provided in Table 5.

**Table 5:** **Univariate (crude) and multivariate (adjusted) analysis of factors predicting hospitalization and DHF at infection two**

	**Odds ratio (95% CI) of hospitalization over non-hospitalization at infection two**		**Odds ratio (95% CI) of DHF over non-DHF at infection two**	
	**Crude**	**Adjusted**	**Crude**	**Adjusted**
Time between infections	1.80*	2.35*	1.62*	3.83*
	(1.21-2.67)	(1.39-3.99)	(1.27-2.06)	(1.71-8.59)
Age at infection one	1.06	1.45	0.97	2.11*
	(0.79-1.40)	(0.97-2.16)	(0.72-1.29)	(1.13-3.93)
Severity at infection one (non-hospitalized as reference)				
DHF	0.87	0.76	2.26	2.70
	(0.19-4.05)	(0.14-4.17)	(0.44-11.66)	(0.39-18.86)
Hospitalized dengue	4.58*	4.22*	5.84*	6.23*
	(1.37-15.24)	(1.18-15.07)	(1.32-25.79)	(1.20-32.40)

*Significant at p < 0.05.

#### Multivariate analysis of association with clinical category

Using a binary logistic regression model, hospitalization at infection two was more strongly associated with time between sequential infections (OR 2.35, 95% CI 1.39-3.99). Hospitalization at infection one continued to pose an increased risk for hospitalization at infection two; DHF and increasing age at infection one did not. Assessing risk factors associated with DHF at infection two, results were consistent with the crude analysis: increasing time between sequential infections and hospitalization at infection one heightened the risk for DHF at infection two, whereas DHF at infection one did not. Variables that were included in the analysis are listed in Table 5.

## Discussion

Using data from over 17 years of hospital-based passive surveillance and nine years of cohort studies, we found 502 individuals with repeat DENV infections among 38,740 DENV infections in our database. In the two cohort studies, a total of 1806 individuals experienced at least one DENV infection; 205 (15.8%) of these individuals were observed to have experienced repeat infections of which 72% of second detected infections were subclinical. This represents one of the largest samples of repeat DENV infections reported to date.

The mean time interval between sequential infections was 3.5 ± 2.4 years. Considering only symptomatic repeat infections, the mean time interval between the first and second detected infections was 4.3 ± 2.4 years, higher than what we reported in 2007 from an analysis of a smaller dataset from QSNICH collected from January 1994 to February 2005 [8]. Similar to Montoya et al. [31], we found that the time from a subclinical infection to a subsequent subclinical infection (1.7 ± 0.8 years) was shorter than to a subsequent symptomatic infection (3.0 ± 2.9 years); p = 0.006.

The shortest duration between sequential symptomatic DENV infections in our study was approximately two months suggesting that heterologous cross-protection lasts for a minimum of two months (although the typical duration may have been longer). This finding is consistent with the serial experimental inoculations of DENV into human volunteers in the 1940s by Sabin [11]. It is possible that the period of complete cross-protection varies according to each particular sequential serotype pair. Despite our relatively large dataset, the number of symptomatic infections with knownserotype was not large enough to demonstrate significant differences among different serotype pairs. Time intervals between serotype pairs would also be affected by the timing of particular serotype predominance in these communities during the 17-year study period, which would represent a confounding factor in this analysis.

Analysis of serologic responses in symptomatic infections revealed that, for 84 individuals, infection one was in fact their first DENV infection, indicating that infection two was their second DENV infection. In contrast, a secondary response in infection one may have represented a first, second, or third DENV infection since a secondary serologic response at the first infection could theoretically have been due to prior Japanese encephalitis virus (JEV) vaccination or natural JEV exposure. Serologic response was not included in our quantitative analysis, however, because a large portion of the cohort extended dataset consisted of subclinical infections for which this information was not available. Instead, we included age at infection one to adjust for the outcome’s association with serologic response, presuming that older individuals were more likely to have had prior DENV infections.

Similar to our previous report in which we found a longer interval between sequential infections to be associated with symptomatic rather than subclinical infection at infection two, both crude and adjusted odds ratios of the cohort extended dataset demonstrated that a longer interval between sequential infections increased the risk of hospitalization and DHF at infection two. Other studies have similarly found that the time between sequential infections is important in determining the severity of the later infection [6,8,30].

We found that hospitalization at the first detected DENV infection was associated with an increased risk for hospitalization and DHF at the second detected infection. Although DHF at infection one did not similarly show a significant association, this may have been due to the limited number of DHF cases. These findings support the notion of individual genetic predisposition for more severe dengue disease. Other studies have found associations between specific HLA classes and disease severity [32-34]; a summary of such associations has been published by Stephens et al. [32]. Other genetic associations have been reported between DF and DHF such as those relevant to TNF-α, iNOS or p47^phox^ [35-37]. Interestingly, among those individuals who had three sequential infections detected in our study (Table 2), two (patients 2 and 5) had subclinical infection with all three infections and two (patients 1 and 8) had DHF with the third infection, suggesting some degree of consistency in severity among some individuals across their infections. Nevertheless, it is also possible that parents of children with previous dengue admissions or DHF may have been more likely to bring their child to medical attention during subsequent episodes.

Our study has several limitations. First, hospital-based passive surveillance would not have identified subclinical and non-hospitalized symptomatic infections. The ability to detect subclinical and mild infections was constrained to relatively short time periods and small groups of individuals in cohort studies. Therefore, analyses of the full dataset may have been confounded when evaluating, for example, the time between sequential infections which may have been underestimated for mild infections. Furthermore, confounders in the analysis of the cohort extended dataset may not have been equally distributed over the whole analysis period due to the inclusion of non-study hospital-based passive surveillance data. Second, the timing of subclinical infection could not be determined definitively and was instead estimated at the midpoint between serial scheduled blood collections. This approximation may have affected our analyses of time between sequential infections in an unpredictable manner. Third, in the hospital-based passive surveillance, the chance of a patient revisiting the same hospital was less likely in Bangkok than KPP given the greater abundance of healthcare facilities in Bangkok. So patients admitted twice to QSNICH may have been more likely to have severe disease with both sequential infections.

## Conclusions

Our study provides a unique opportunity to evaluate a large number of repeat DENV infections over an extended period of time. This was made possible by the long-standing dengue testing that has been performed at the same locations in Thailand over many years. Our findings provide further evidence of the importance of heterologous cross-protection and the possibility of genetic predisposition in determining the clinical presentation of DENV infection. These findings should inform ongoing investigations of DENV transmission dynamics, development of vaccines and dengue biomarkers.

## Additional file

Additional file 1:
**Comparison between (1) additional individuals found to have repeat dengue virus infections by evaluating the cohort extended dataset, and (2) individuals found to have repeat infections solely by using cohort study data.**
