# Supplementary material for: Sequential dengue virus infections detected in active and passive surveillance programs in Thailand, 1994–2010
Source: BMC Public Health. 2015 Mar 14;15:250. doi: 10.1186/s12889-015-1590-z (PMC4371716; doi:10.1186/s12889-015-1590-z)
Supplement: Additional file 1: — Comparison between (1) additional individuals found to have repeat dengue virus infections by evaluating the cohort extended dataset, and (2) individuals found to have repeat infections solely by using cohort study data. [file 12889_2015_1590_MOESM1_ESM.pdf]

Additional file 1: Comparison between (1) additional individuals found to have sequential dengue infections by extending cohort follow-up using passive hospital-based surveillance, opposed to (2) individuals found by solely using data within the cohort.

Data shown below are aimed provide a better picture of the possible bias introduced into the analyses by adding data of individuals with sequential dengue captured not solely from cohort study activities. As expected, distribution of clinical manifestation between the two data groups (1) and (2) were not similar; (2) highly skewed towards subclinical, whereas (1) showed skewness to DHF at infection one and more evenly distributed at infection two.

	**(1) Cohort enrolled with extended follow-up (N=37)**		**(2) Solely captured from cohort (N=158)**	
	N	%	N	%
**Gender**				
Female	24	65	74	47
Male	13	35	74	47
Unknown	-	-	10	6
**Clinical manifestation at infection one**				
Subclinical	5	14	74	47
Subclinical/non-hospitalized	3	8	53	34
Symp. (hosp./non-hosp. DF)	-	-	3	2
Symp. (non-hospitalized DF)	1	3	18	11
Symp. (hospitalized DF)	10	27	4	3
Symp. (DHF/hospitalized DF)	1	3	2	1
Symp. (DHF)	17	46	4	3
**Clinical manifestation at infection two**				
Subclinical	10	27	90	57
Subclinical/non-hospitalized	8	22	34	22
Symp. (hosp./non-hosp. DF)	-	-	1	1
Symp. (non-hospitalized DF)	6	16	22	14
Symp. (hospitalized DF)	3	8	9	6
Symp. (DHF/hospitalized DF)	-	-	-	-
Symp. (DHF)	10	27	2	1

Sequential pattern of clinical manifestation introduced by the extended group (1) mostly provided patterns of severe followed by less severe and less-severe followed by more severe. The continuingly severe followed by severe pattern is limited. In contrast, the group from solely cohort data (2) was highly populated in the subclinical followed by subclinical segment.

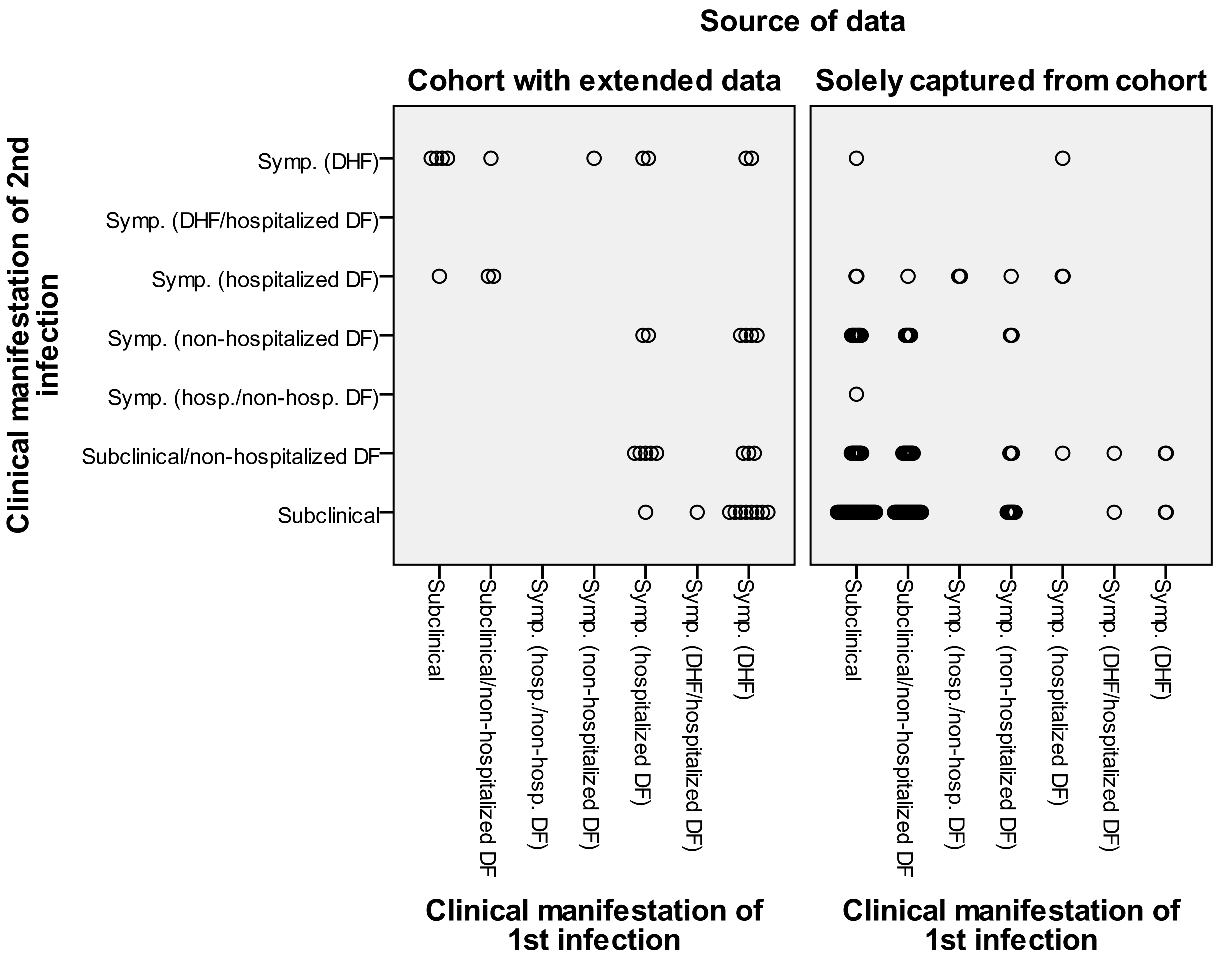

Age distribution in group (1) was close to evenly distributed at infection one and two. Group (2) performed a right-tailed skew (at infection one) and a left-tailed skew (at infection two); this reflected the lessened chance of detection for subjects enrolled late after study activity had started.

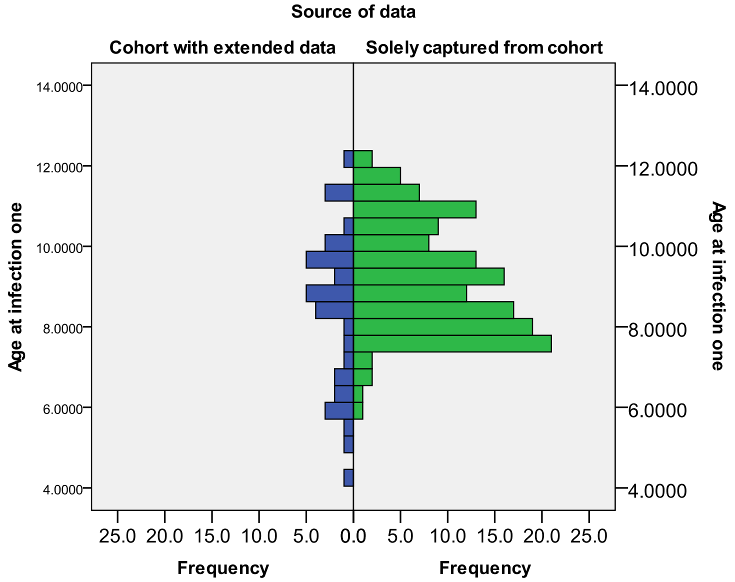

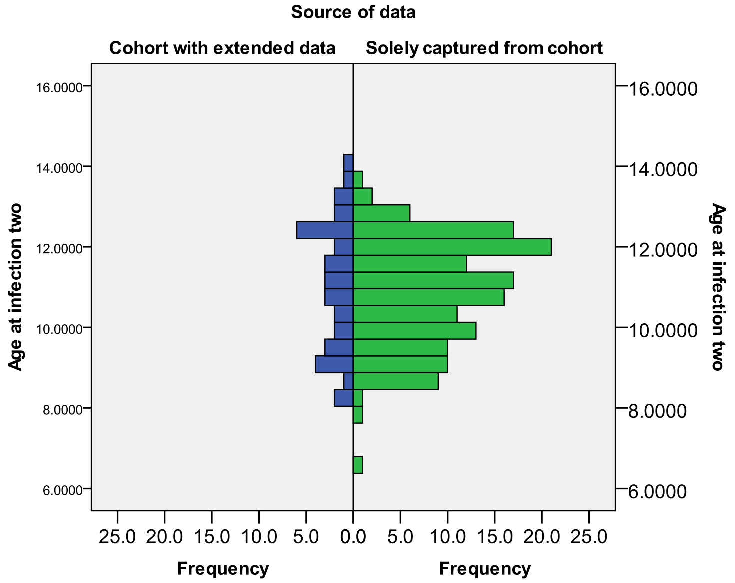

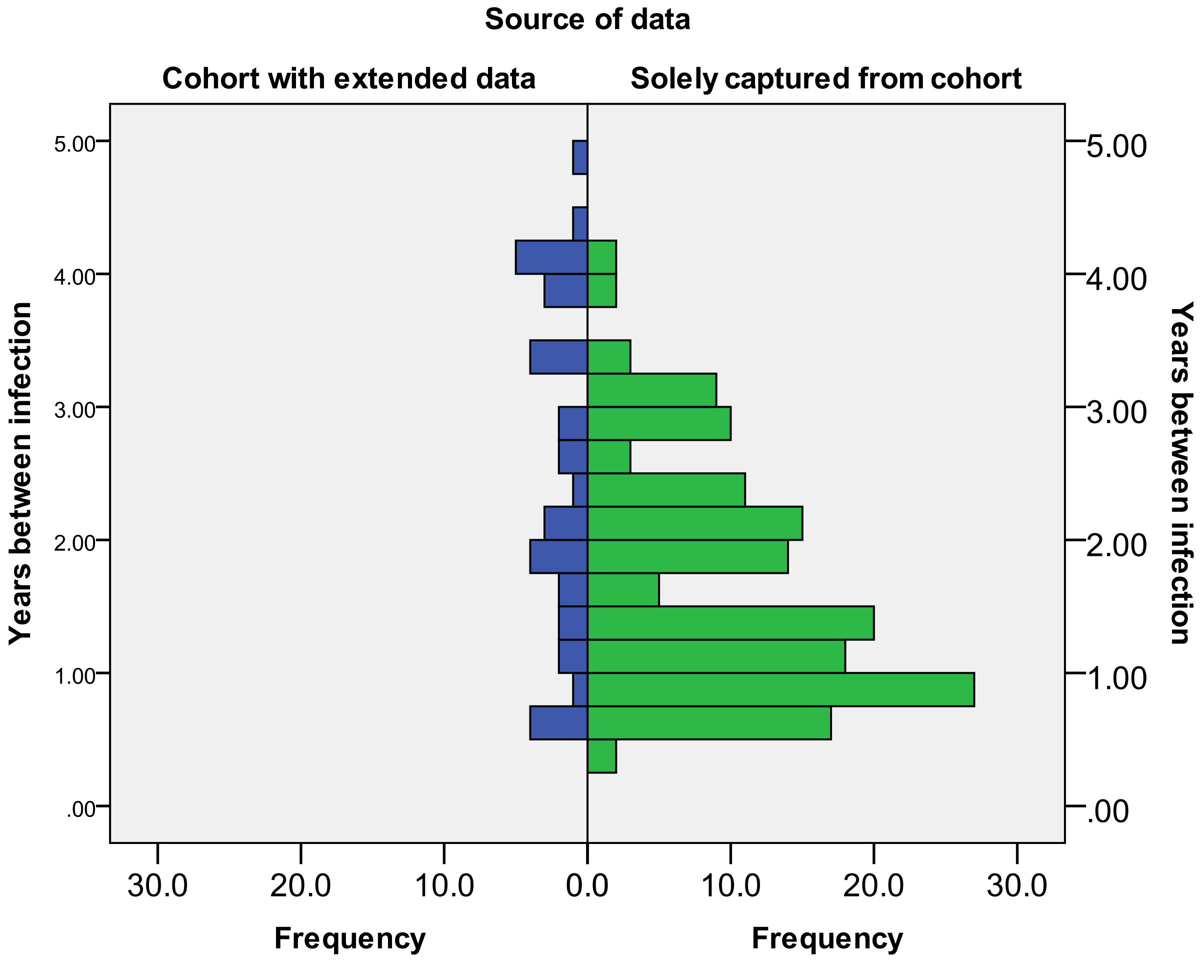
Source of data
Cohort with extended data
Solely captured from cohort
Years between infection
5.00
4.00
3.00
2.00
1.00
.00
30.0
20.0
10.0
0.0
Frequency
